# Albumin levels in malaria patients: a systematic review and meta-analysis of their association with disease severity

**DOI:** 10.1038/s41598-024-60644-z

**Published:** 2024-05-03

**Authors:** Saruda Kuraeiad, Kwuntida Uthaisar Kotepui, Aongart Mahittikorn, Frederick Ramirez Masangkay, Polrat Wilairatana, Apiporn Thinkhamrop Suwannatrai, Kavin Thinkhamrop, Kinley Wangdi, Manas Kotepui

**Affiliations:** 1 Medical Technology, School of Allied Health Sciences, Walailak University, Tha Sala, Nakhon Si Thammarat, 80160 Thailand; 2 Department of Protozoology, Faculty of Tropical Medicine, Mahidol University, Bangkok, 10400 Thailand; 3 Department of Medical Technology, Faculty of Pharmacy, University of Santo Tomas, 1008 Manila, Philippines; 4 Department of Clinical Tropical Medicine, Faculty of Tropical Medicine, Mahidol University, Bangkok, 10400 Thailand; 5 Department of Parasitology, Faculty of Medicine, Khon Kaen University, Khon Kaen, 40002 Thailand; 6 Faculty of Public Health, Khon Kaen University, Khon Kaen, 40002 Thailand; 7 Health Research Institute, University of Canberra, Bruce, ACT 2601 Australia; 8 QIMR Medical Research Institute, 300 Herston Road, Herston, QLD 4006 Australia; 9 College of Health and Medicine, Australian National University, Acton, ACT 2601 Australia; 10 Research Center in Tropical Pathobiology, Walailak University, Nakhon Si Thammarat 80160, Thailand; 11 Medical Technology Program, Faculty of Science, Nakhon Phanom University, Nakhon Phanom 48000, Thailand

**Keywords:** Albumin, Malaria, *Plasmodium*, Severity, Complications, Clinical biochemistry, Malaria, Diagnostic markers, Prognostic markers

## Abstract

Albumin, a key protein in human blood plasma, has been linked to various health conditions. However, its association with malaria, particularly in assessing disease severity, remains inadequately understood. This comprehensive systematic review and meta-analysis aimed to elucidate the relationship between albumin levels and malaria severity. A comprehensive literature search was conducted across multiple databases, including Embase, Scopus, PubMed, MEDLINE, Ovid, and Google Scholar, to identify studies examining albumin levels in malaria patients. The Preferred Reporting Items for Systematic Reviews and Meta-Analyses (PRISMA) guidelines were followed. Data were pooled using a random-effects model, and heterogeneity was assessed using *I*^2^ statistics. Subgroup and meta-regression analyses were performed based on publication year, study location, and *Plasmodium* species. A total of 37 studies were included in this review. The thematic synthesis indicated that albumin levels in malaria patients varied significantly based on geographical location. A meta-analysis of 28 studies found that albumin levels were significantly lower in malaria patients compared with non-malarial controls (*P* < 0.001, standardized mean differences [SMD] = −2.23, 95% CI − 3.25 to − 1.20, *I*^2^: 98%, random effects model, 28 studies). Additionally, subgroup analysis revealed variations in albumin levels based on geographical location and *Plasmodium* species. Regarding the association with disease severity, thematic synthesis showed that severe malaria cases generally had decreased albumin levels across various regions. However, one Brazilian study reported higher albumin levels in severe cases. A separate meta-analysis of five studies found significantly lower albumin levels in patients experiencing severe malaria relative to those with less severe forms of the disease (*P* < 0.001, SMD = −0.66, 95% CI − 1.07 to − 0.25), *I*^2^: 73%, random effects model, 5 studies). This study underscores the clinical significance of albumin as a potential biomarker for *Plasmodium* infection and the severity of malaria. The findings suggest that albumin level monitoring could be crucial in managing malaria patients, especially in assessing disease severity and tailoring treatment approaches. Additional studies are required to investigate the underlying mechanisms driving these associations and validate the clinical utility of albumin levels in malaria patient management.

## Introduction

Malaria is a life-threatening infectious disease caused by *Plasmodium* parasites transmitted to people through the bites of infected female *Anopheles* mosquitoes^1^. It is a major health problem in tropical and subtropical regions of the world, particularly in sub-Saharan Africa and South Asia^2^. Malaria in humans is attributable to infection by five distinct species of the *Plasmodium* parasite: *P. falciparum*, *P. vivax*, *P. ovale*, *P. malariae*, and *P. knowlesi*. Of these, *P. falciparum* poses the greatest risk and is Africa's most encountered variant^2^. Malaria can present with a range of symptoms, from asymptomatic to severe complications, and may lead to death if left untreated^1^. The most severe and deadly form is primarily caused by *P. falciparum*, with fewer cases attributed to *P. vivax*^3–5^ and other *Plasmodium* species, including zoonotic malaria caused by *P. knowlesi*^6–8^. Although preventive measures such as mosquito nets and insecticides, along with treatment protocols involving antimalarial medications, have been implemented to reduce malaria incidence and fatalities, the disease continues to significantly impact public health and economies in endemic areas.

Albumin, a protein synthesized by the liver, is the most abundant protein in the blood plasma of humans and other vertebrates^9^. It plays several vital roles, including maintaining oncotic pressure, crucial for fluid distribution and balance in the body^9^. Additionally, albumin is a carrier for various substances in the blood, such as hormones, vitamins, and drugs, and is involved in tissue growth and healing^10,11^. The blood level of albumin reflects liver function and nutritional status and is used to assess the severity of various diseases, including cardiovascular disease^12,13^, renal diseases^14,15^, or ulcerative colitis^16^. While the relationship between malaria and albumin levels remains unclear, it is essential to consider the lifecycle of the malaria parasite, particularly its replication in the liver^17,18^, to elucidate this connection. The liver is instrumental in albumin synthesis, and the malaria parasite's replication within the liver could impact its function and, subsequently, albumin production and regulation. In severe *P. falciparum* infections, hypoalbuminemia, or low albumin levels, have been observed and linked to acute renal failure^19^. Furthermore, hypoalbuminemia has been associated with the development of shock in adults with severe *P. falciparum* infections^20^. The present systematic review and meta-analysis aim to determine the difference in albumin levels between those with and without malaria and between severe and non-severe malaria cases. The results of this study could provide helpful information to enhance early detection of severe cases, inform treatment decisions, and potentially lead to interventions like albumin supplementation. Additionally, the findings may offer a deeper understanding of the disease’s pathophysiology, guide future research, and shape public health policies, especially in regions where malaria is endemic.

## Methods

### Protocol and registration

The protocol of the systematic review and meta-analysis was registered in PROSPERO (CRD42023471881). The results were reported according to the Preferred Reporting Items for Systematic Reviews and Meta-Analyses (PRISMA) guidelines^21^.

### Systematic review question

The review questions were guided by the Population, Exposure, Comparator, Outcome (PECO) framework^22^. The population (P) consisted of participants in malaria-endemic areas; the exposure (E) was *Plasmodium* infection or severe malaria; the comparator (C) was non-malarial controls or non-severe malaria cases; the outcome (O) was blood albumin levels.

### Search strategy and selection criteria

A comprehensive literature search was conducted across multiple databases, including Embase, Scopus, PubMed, MEDLINE, and Ovid, to identify studies pertaining to albumin levels in malaria patients. The search strategy incorporated a range of terms and combinations related to "malaria" and "albumin," with the general search string being “albumin AND (malaria OR *plasmodium* OR '*Plasmodium* infection' OR 'Remittent Fever' OR 'Marsh Fever' OR paludism).” The search strategy varied slightly between databases (Table S1). Studies from the inception of each database up to the present were included without language restrictions. Additional records were identified through a Google Scholar search.

### Study selection and data extraction

Duplicate records from all databases were removed before screening. Titles and abstracts were then screened to identify studies that reported albumin levels in human participants with malaria. Full texts of potentially eligible studies were retrieved and assessed for inclusion. Studies were omitted from consideration if they were in vitro studies, reviews, or did not report albumin levels in malaria patients. Only studies that provided precise comparative data on albumin between malaria patients and non-malarial controls or between different malaria severity levels were included. Study selection was performed independently by two authors (SK, MK), and any disagreements were resolved by consulting a third author (AM).

Data extracted from the included studies were composed of study characteristics (publication year, study design, geographical location), participant demographics (age group, clinical status), the method of *Plasmodium* detection, and the type of blood sample used for albumin testing. One author (MK) performed the data extraction and cross-checked by another author (AM).

### Quality assessment

The Joanna Briggs Institute (JBI) critical appraisal tools were used for the evaluation of cross-sectional, cohort, case–control, and quasi-experimental studies, each tailored to address specific research design intricacies^23^. For cross-sectional studies, the tool focuses on the clarity of criteria for inclusion, the reliability and accuracy of the measures for exposure and outcomes, and the identification and management of confounding variables. The cohort tool evaluates the similarity of cohorts based on their design or analytical approach and the completeness of follow-up. For case–control studies, it scrutinizes the comparability between cases and controls, the methods for case ascertainment, and the control selection process. The tool for quasi-experimental studies examines the integrity of the intervention's implementation, the outcomes' measurements, and the appropriateness of the statistical analysis to control for confounding factors. Quality assessment was performed independently by two authors (SK, MK), and any disagreements were resolved by discussion to arrive at a consensus.

### Data synthesis and analysis

To perform the thematic synthesis, data were extracted from each included study on the differences in albumin levels. This included specific outcomes related to albumin levels in different subpopulations of malaria patients, such as those with varying degrees of malaria severity, geographical differences, and comparison between malaria patients and non-malarial controls. The results from the individual studies were synthesized to construct a narrative that described the overall findings related to albumin levels in malaria patients.

A meta-analysis was conducted to synthesize the data from studies comparing albumin levels between malaria patients and non-malarial controls, and between patients experiencing severe malaria relative to those with less severe forms of the disease. Standardized mean differences (SMD, Hedge’s g) and 95% confidence intervals (CI) were calculated using a random-effects model to account for between-study heterogeneity, which was quantified using the *I*^2^ statistic in which *I*^2^ more than 50% suggest significant heterogeneity^24^. Meta-regression and subgroup analyses were conducted to investigate potential sources of heterogeneity. Explanatory factors considered were the year of publication, design of the study, location, age demographics, *Plasmodium* species, clinical condition, method of diagnosis, and sample type. A cumulative meta-analysis assessed trends over time in the difference in albumin levels.

A funnel plot was constructed to assess publication bias, and the linear regression test for funnel plot asymmetry was used. Sensitivity analysis was carried out to assess the impact of each individual study on the collective results of the meta-analysis. Outlier detection methods were applied to identify and exclude studies that significantly deviated from the overall effect estimate. A power analysis was performed to determine if the number of included studies was sufficient to detect a significant difference in albumin levels. The statistical analysis was conducted using RStudio (Version: 2023.09.1+494)^25^.

## Results

### Search results

**Figure 1:**
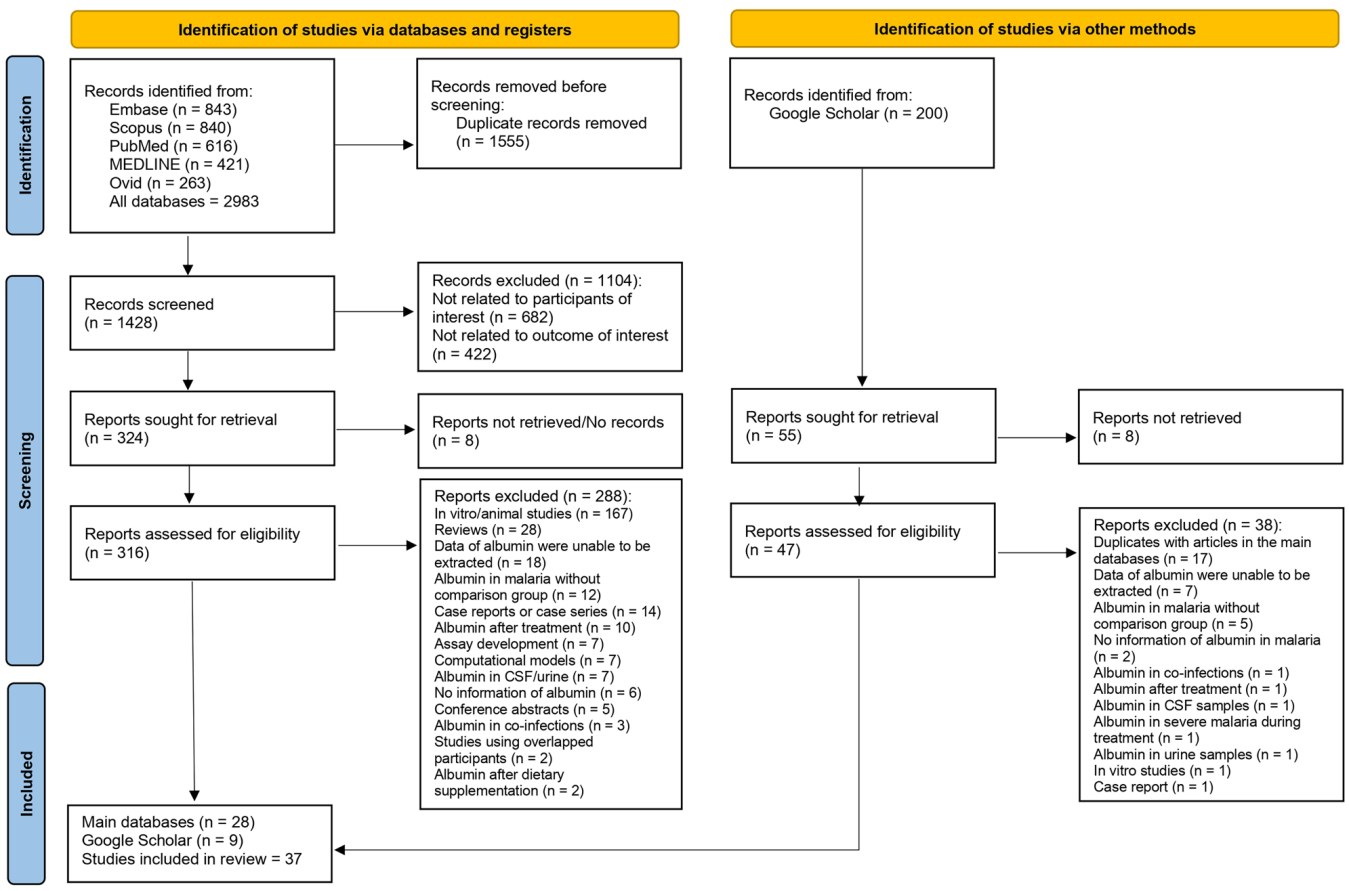
Study flow diagram. The study diagram shows the steps of study selection from the main databases and Google Scholar.

From the databases, 2983 records were identified: 843 from Embase, 840 from Scopus, 616 from PubMed, 421 from MEDLINE, and 263 from Ovid. Before screening, 1555 duplicates were removed, leaving 1428 records. Of these, 1104 were excluded for not relating to participants or the outcome of interest. Retrieval was sought for 324 reports, but 8 could not be retrieved. A total of 316 reports underwent eligibility assessment, and 288 were excluded for several factors, including their nature as in vitro studies, reviews, or lacking pertinent data on albumin. From the main databases, there were 28 records and nine studies were from Google Scholar totaling 37 studies for the review (Fig. 1).

### Characteristics of studies

Of the 37 studies included, nearly half were published between 2010 and 2019 (43.2%) and predominantly used a case–control design (51.4%). The studies were mainly conducted in Asia (43.2%) and Africa (46.0%). The majority focused on *P. falciparum* (73.0%). In terms of participants, adults (46.0%) were most studied, followed by children (27.0%). Symptomatic malaria was the primary symptom under investigation in 67.6% of the studies. The microscopic method was the most prevalent *Plasmodium* detection method (70.3%), and serum was the most used blood sample for albumin testing (59.5%) (Table 1; Table S2).

**Table 1:** Descriptive characteristics of the 37 studies included in the systematic review and meta-analysis.

Characteristics	n. (37 studies)	%
Publication year		
Before 2000	10	27.03
2000–2009	6	16.22
2010–2019	16	43.24
2020–2023	5	13.51
Study designs		
Case–control study	19	51.35
Cross-sectional study	11	29.73
Cohort study	6	16.22
Quasi-experimental study	1	2.70
Study areas		
Asia	16	43.24
India	8	27.62
Thailand	4	10.81
Turkey	2	5.41
Indonesia	1	2.70
Lao People’s Democratic Republic	1	2.70
Africa	17	45.95
Nigeria	11	29.73
Sudan	2	5.41
Gambia	1	2.70
Ethiopia	1	2.70
Cameroon	1	2.70
Uganda	1	2.70
Europe	2	5.41
France	1	2.70
Germany	1	2.70
Oceania (Papua New Guinea)	1	2.70
South America (Brazil)	1	2.70
*Plasmodium* species		
* P. falciparum*	27	72.97
* P. vivax*	2	5.41
* P. falciparum*, *P. vivax*	1	2.70
Not specified	7	18.92
Participants		
Adults	17	45.95
Children	10	27.03
Children and adults	6	16.22
Not specified	4	10.81
Symptom		
Symptomatic malaria	25	67.57
Asymptomatic malaria	1	2.70
Symptomatic and asymptomatic malaria	2	5.41
Not specified	9	24.32
Methods for *Plasmodium* detection		
Microscopic method	26	70.27
Microscopic method, RDT	5	13.51
RDT	1	2.70
Not specified	5	13.51
Blood sample for albumin		
Serum	22	59.46
Plasma	12	32.43
Not specified	3	8.11

*RDT* rapid diagnostic test.

### Quality of the included studies

In the evaluation of analytical cross-sectional studies using the JBI critical appraisal checklist (Table S2), several studies clearly defined their inclusion criteria, detailed their study subjects and settings, and used valid and reliable measures for both exposure and outcomes^26–36^. However, the identification of confounding factors and strategies to deal with them were often missing or unclear. In the JBI critical appraisal checklist for case–control studies, several studies generally demonstrated a robust methodology, with all ensuring comparability of groups, appropriate matching, and standard criteria for identification^37–55^. Most studies measured exposure consistently, yet some had unclear aspects in exposure measurement and outcome assessment. A common gap identified was the lack of approaches to address potential confounders.

In the JBI critical appraisal checklist for cohort studies, all studies recruited groups from the same population and measured exposures in a similar manner^56–61^. Each study ensured that the exposure was measured in a valid and reliable way and that participants were free of the outcome at the study's start. The follow-up time was reported to be sufficient for the occurrence of outcomes. However, not all studies identified or stated strategies to deal with confounding factors. Moreover, some studies did not adequately address follow-up completeness or strategies for incomplete follow-up^56,60^. The quasi-experimental study clearly defined all criteria related to the JBI critical appraisal checklist^62^.

### Thematic synthesis

**Figure 2:**
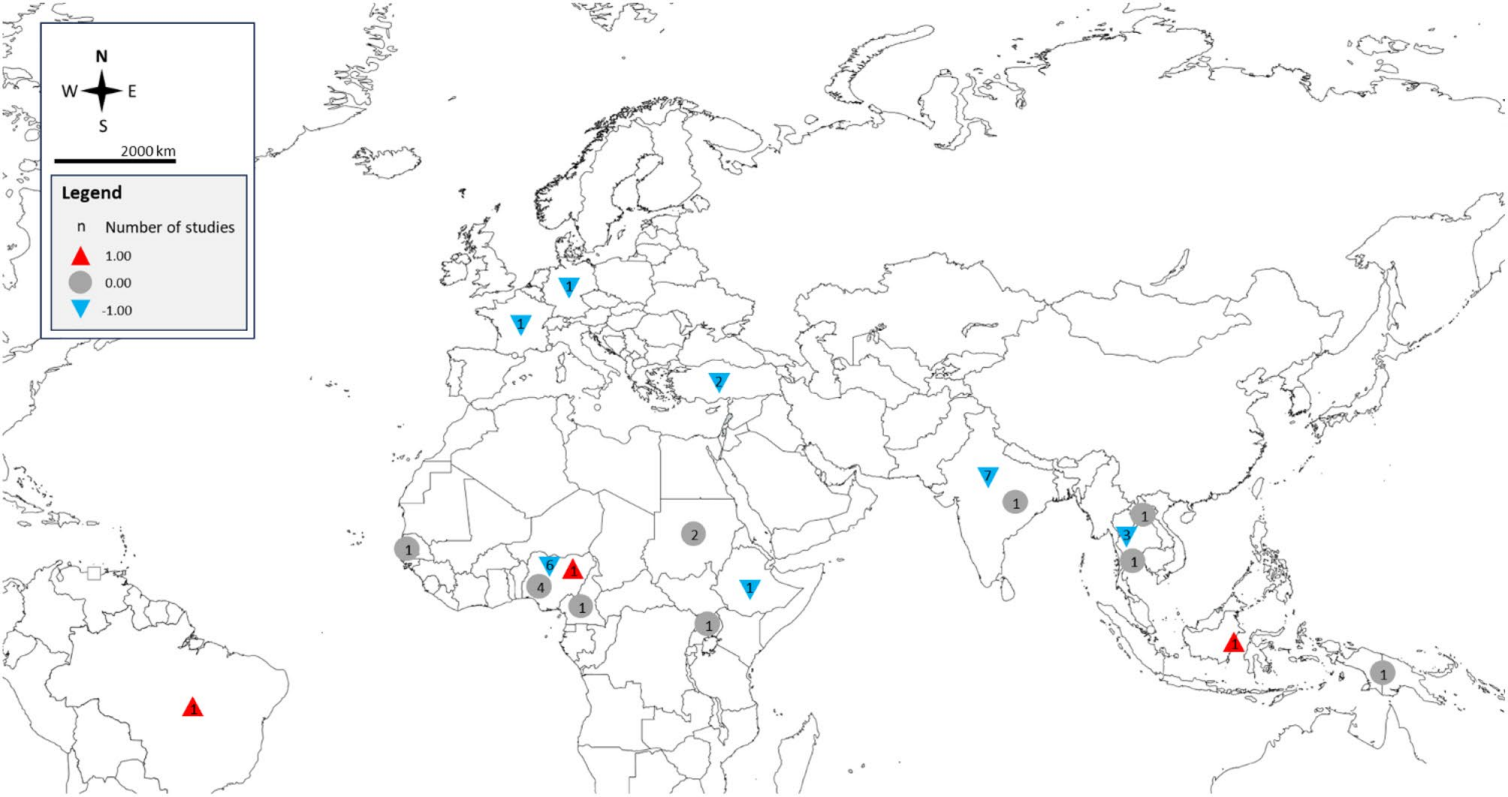
Country distribution of albumin level changes in patients with malaria. Numbers in symbols (red triangle, gray circle, blue triangle) are the number of studies. Red triangles represent studies reporting increased albumin levels in malaria patients compared to non-malaria patients. Blue triangles represent studies reporting decreased albumin levels in malaria patients compared to non-malaria patients. Gray circles represent studies reporting no difference in albumin levels between malaria patients and non-malaria patients. Map template sourced from mapchart.net.

Of the 37 studies included in the review (Table 2), 36 compared and presented the differences in albumin levels between malaria patients and non-malarial controls or between patients experiencing severe malaria relative to those with less severe forms of the disease^26–45,47–62^ (Fig. 2). However, Davis et al. did not specify or present the results of the variation in albumin levels across the two groups^46^. Studies from various regions within Africa reported mixed outcomes regarding albumin levels in malaria patients compared to non-malarial controls. A number of studies indicated no notable disparity in albumin levels when comparing malaria patients with non-malarial controls, reflecting a diverse array of findings across the continent^34,37,38,49,52,58,61^. Conversely, studies from sub-Saharan Africa observed a reduction in albumin levels among malaria patients, suggesting a potential association between malaria infection and decreased albumin levels in this region^26,29,32,39,42,47,62^. Moreover, a distinct finding from Nigeria, within the West African sub-region, identified increased albumin levels in malaria patients^51^. Specifically, Olukemi et al. from Nigeria found no significant difference in albumin levels between patients with mild parasitemia and non-malarial controls. However, there was a noticeable reduction in albumin levels among individuals with moderate parasitemia compared to those with no parasitemia^35^. This study also underscored an inverse relationship between albumin levels and the degree of parasitemia. Pankoui Mfonkeu et al. indicated no substantial variation in uncomplicated malaria patients and non-malarial controls. Yet, a significant decrease was observed in cerebral malaria patients (excluding those with malaria anemia) relative to non-malarial controls^36^. Studies conducted by Akiyama T et al. and Bhattacharjee et al. from Asia revealed no notable variation in albumin levels when comparing malaria patients to those without malaria^27,43^; and decreased albumin levels in malaria patients^28,33,40,41,44,45,48,54,55,57,59^. In Oceania, specifically Papua New Guinea, no significant variation was observed in albumin levels between malaria patients and individuals without malaria^50^.

**Table 2:** Summary of individual study findings on albumin levels in malaria patients.

No	References	Continent	Country	*Plasmodium* spp.	Age range (years)	Clinical malaria (symptomatic or asymptomatic)	Comparison	Results of individual study
1	Abdagalil et al.^37^	Africa	Sudan	*P. falciparum*	20–40	Not specified	Malaria patients vs. uninfected controls	No significant difference in albumin levels between malaria patients and uninfected controls
2	Adamu et al.^38^	Africa	Nigeria	Not specified	1–50	Not specified	Malaria patients vs. uninfected controls	No significant difference in albumin levels between malaria patients and uninfected controls
3	Adeosun et al.^26^	Africa	Nigeria	*P. falciparum*	1–10	Symptomatic malaria	Malaria patients vs. uninfected controls Association between albumin and parasite density	1. Albumin levels were significantly decreased in malaria patients compared to uninfected controls 2. No significant difference in albumin levels between high and low parasite density
4	Akiyama et al.^27^	Asia	Lao People’s Democratic Republic	*P. falciparum*	Patients with malaria (40): 4–45, non-malaria (31): 5–55	Not specified	Malaria patients vs. uninfected controls	No significant difference in albumin levels between malaria patients and uninfected controls
5	Amah et al.^39^	Africa	Nigeria	*P. falciparum*	30–65	Not specified	Malaria patients vs. uninfected controls	Albumin levels were significantly decreased in malaria patients compared to uninfected controls
6	Areekul et al.^40^	Asia	Thailand	*P. falciparum*	18–45	Symptomatic malaria	Malaria patients vs. uninfected controls	Albumin levels were significantly decreased in malaria patients compared to uninfected controls
7	Ayyadevara et al.^41^	Asia	India	*P. falciparum*, *P. vivax*	Not specified	Symptomatic malaria	Malaria patients vs. uninfected controls	Albumin levels were significantly decreased in malaria patients compared to uninfected controls
8	Balogun et al.^42^	Africa	Nigeria	Not specified	15–40	Symptomatic malaria	Malaria patients vs. uninfected controls Association between albumin and parasite density	1. Albumin levels were significantly decreased in malaria patients compared to uninfected controls 2. No significant difference in albumin levels between high and low parasite density
9	Bhattacharjee et al.^43^	Asia	India	*P. falciparum*	Not specified	Not specified	Malaria patients vs. uninfected controls	No significant difference in albumin levels between malaria patients and uninfected controls
10	Bruneel et al.^56^	Europe	France	*P. falciparum*	Not specified	Symptomatic malaria	Severe vs. less severe malaria	Albumin levels were significantly decreased in severe malaria compared to uncomplicated malaria
11	Camacho et al.^57^	Asia	Thailand	*P. falciparum*	Not specified	Symptomatic malaria	Severe vs. less severe malaria	Albumin levels were significantly decreased in severe malaria compared to uncomplicated malaria
12	Conroy et al.^58^	Africa	Uganda	*P. falciparum*	1.5–12	Symptomatic malaria	Malaria patients vs. uninfected controls Cerebral vs. severe malarial anemia	1. No significant difference in albumin levels between malaria patients and uninfected controls 2. No significant difference in albumin levels between cerebral malaria and severe malarial anemia
13	Das et al.^44^	Asia	India	*P. falciparum*	Not specified	Symptomatic malaria	Malaria patients vs. uninfected controls	Albumin levels were significantly decreased in malaria patients compared to uninfected controls
14	Das et al.^59^	Asia	India	*P. falciparum*	2–12	Not specified	Malaria patients vs. uninfected controls	Albumin levels were significantly decreased in malaria patients compared to uninfected controls
15	Das et al.^45^	Asia	India	*P. falciparum*	2–11	Symptomatic malaria	Malaria patients vs. uninfected controls Severe vs. less severe malaria	1. Albumin levels were significantly decreased in malaria patients compared to uninfected controls 2. Albumin levels were significantly decreased in severe malaria compared to mild malaria 3. No difference in albumin levels between mild malaria and asymptomatic malaria 4. Albumin levels were significantly decreased in severe malaria compared to asymptomatic malaria
16	Davis et al.^46^	Asia	Thailand	*P. falciparum*	14–49	Symptomatic malaria	Severe vs. less severe malaria	Did not specify comparative results. Quantitative albumin levels: Severe malaria (n = 18): median 2.40 (2.20–2.90), uncomplicated malaria (n = 14): median 2.68 (2.40–3.15) g/dl
17	Devi et al.^28^	Asia	India	Not specified	Not specified	Symptomatic and asymptomatic malaria	Malaria patients vs. uninfected controls	Albumin levels were significantly decreased in malaria patients compared to uninfected controls (healthy and febrile controls)
18	Ebrahim et al.^47^	Africa	Ethiopia	*P. falciparum*	Not specified	Asymptomatic malaria	Malaria patients vs. uninfected controls Association between albumin and parasite density	1. Albumin levels were significantly decreased in malaria patients compared to uninfected controls 2. Albumin levels were significantly decreased in high parasitemia compared to moderate and low parasitemia
19	Erel et al.^48^	Asia	Turkey	*P. vivax*	15–35	Not specified	Malaria patients vs. uninfected controls	Albumin levels were significantly decreased in malaria patients compared to uninfected controls
20	Etim et al.^62^	Africa	Nigeria	*P. falciparum*	20–55	Not specified	Malaria patients vs. uninfected controls	Albumin levels were significantly decreased in malaria patients compared to uninfected controls
21	Fisayo et al.^29^	Africa	Nigeria	Not specified	Not specified	Symptomatic malaria	Malaria patients vs. uninfected controls	Albumin levels were significantly decreased in malaria patients compared to uninfected controls
22	Fitri et al.^30^	Asia	Indonesia	*P. falciparum*	Not specified	Symptomatic malaria	Severe vs. less severe malaria	Albumin levels were significantly increased in severe malaria compared to uncomplicated malaria
23	Graninger et al.^60^	South America	Brazil	*P. falciparum*	16–50	Symptomatic malaria	Severe vs. less severe malaria	Albumin levels were significantly increased in severe malaria compared to uncomplicated malaria
24	Hoffmeister et al.^31^	Europe	Germany	*P. falciparum*	Not specified	Symptomatic malaria	Severe vs. less severe malaria	Albumin levels were significantly decreased in severe malaria compared to uncomplicated malaria
25	Kayode et al.^32^	Africa	Nigeria	*P. falciparum*	14–30	Symptomatic and asymptomatic malaria	Malaria patients vs. uninfected controls	Albumin levels were significantly decreased in malaria patients compared to uninfected controls
26	Mohanty et al.^33^	Asia	India	*P. falciparum*	Not specified	Symptomatic malaria	Malaria patients vs. uninfected controls Severe vs. less severe malaria	1. Albumin levels were significantly decreased in malaria patients compared to uninfected controls 2. Albumin levels were significantly decreased in severe malaria compared to mild malaria
27	Nsonwu-Anyanwu et al.^49^	Africa	Nigeria	*P. falciparum*	1–15	Symptomatic malaria	Malaria patients vs. uninfected controls Severe vs. less severe malaria	1. No significant difference in albumin levels between malaria patients and uninfected controls 2. Albumin levels were significantly decreased in severe malaria compared to mild malaria
28	O’Donnell et al.^50^	Oceania	Papua New Guinea	*P. falciparum*	Not specified	Symptomatic malaria	Malaria patients vs. uninfected controls Severe vs. less severe malaria	1. No significant difference in albumin levels between malaria patients and uninfected controls 2. Albumin levels were significantly decreased in severe malaria compared to non-severe malaria
29	Ogbodo et al.^51^	Africa	Nigeria	Not specified	5–12	Symptomatic malaria	Malaria patients vs. uninfected controls Association between albumin and parasite density	1. Albumin levels were significantly increased in malaria patients with low and moderate parasite density compared to uninfected controls 2. No significant difference in albumin levels between malaria patients with high parasite density compared to uninfected controls
30	Okon et al.^34^	Africa	Nigeria	*P. falciparum*	Not specified	Symptomatic malaria	Malaria patients vs. uninfected controls	No significant difference in albumin levels between malaria patients and uninfected controls
31	Olukemi et al.^35^	Africa	Nigeria	*P. falciparum*	20–39	Symptomatic malaria	Malaria patients vs. uninfected controls Association between albumin and parasite density	1. No significant difference in albumin levels between mild parasitemia and uninfected controls 2. Albumin levels were significantly decreased in moderate parasitemia compared to no parasitemia 3. Albumin levels were increased as the level of parasitaemia increased
32	Pankoui Mfonkeu et al.^36^	Africa	Cameroon	*P. falciparum*	Uncomplicated malaria (94): 6–168 months, malaria anemia (73): 7–156 months, cerebral malaria (45): 6–134 months, cerebral malaria/malaria anemia (13): 9–96 months, children who had come for vaccination or counseling (45 control): 6–156 months	Symptomatic malaria	Malaria patients vs. uninfected controls	1. No significant difference in albumin levels between uncomplicated malaria patients and uninfected controls 2. Albumin levels were significantly decreased in cerebral malaria (but not malaria anemia) compared to uninfected controls
33	Saad et al.^52^	Africa	Sudan	*P. falciparum*	Not specified	Not specified	Malaria patients vs. uninfected controls Severe vs. less severe malaria	1. No significant difference in albumin levels between malaria patients and uninfected controls 2. No significant difference in albumin levels between severe malaria and uncomplicated malaria
34	Sagaki et al.^53^	Asia	Thailand	*P. falciparum*	> 15	Symptomatic malaria	Severe vs. less severe malaria	Albumin levels were significantly decreased in severe malaria compared to non-severe malaria
35	Seyrek et al.^54^	Asia	Turkey	*P. vivax*	10–32	Symptomatic malaria	Malaria patients vs. uninfected controls	Albumin levels were significantly decreased in malaria patients compared to uninfected controls
36	Snow et al.^61^	Africa	Gambia	Not specified	1–9	Symptomatic malaria	Malaria patients vs. uninfected controls	No significant difference in albumin levels between malaria patients and uninfected controls
37	Umeshchandra et al.^55^	Asia	India	Not specified	Not specified	Symptomatic malaria	Malaria patients vs. uninfected controls	Albumin levels were significantly decreased in malaria patients compared to uninfected controls

When comparing albumin levels between patients experiencing severe malaria relative to those with less severe forms of the disease, the African study conducted by Nsonwu-Anyanwu et al. demonstrated significantly decreased albumin levels in severe malaria compared to mild malaria^49^. However, Saad et al. indicated no notable disparity between severe and uncomplicated malaria patients^52^. Asian studies showed notable reduction in albumin levels among severe malaria patients compared to those with mild malaria^30,33,45,57^. Sagaki et al. conducted the study in Thailand also found decreased levels of albumin in patients experiencing severe malaria relative to those with less severe forms of the disease^53^. Studies by Bruneel F et al. and Hoffmeister et al. from Europe, specifically from France^56^, and Germany^31^, indicated that albumin levels were significantly lower in severe malaria patients compared to those with uncomplicated malaria. A study conducted by Graninger et al. in Brazil highlighted that albumin levels were significantly higher in severe malaria patients compared to those with uncomplicated malaria^60^.

### Meta-analysis

**Figure 3:**
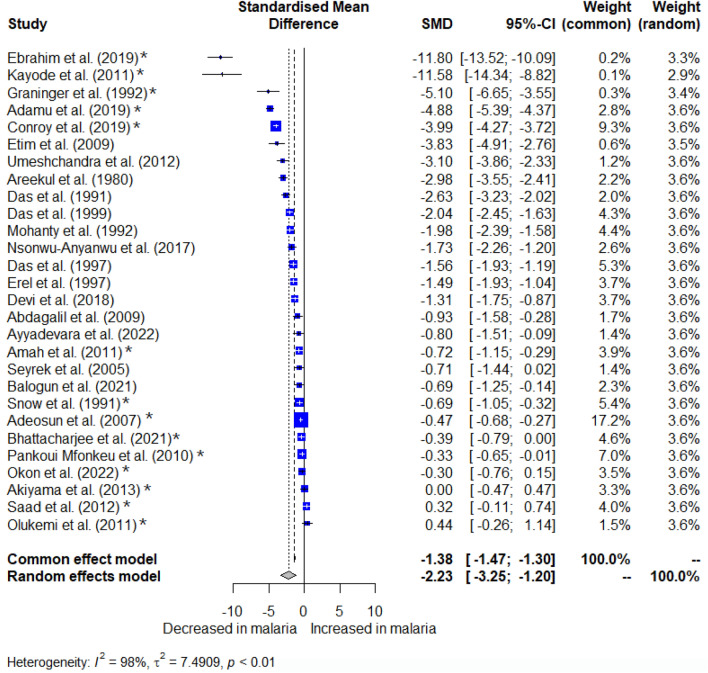
Forest plot displaying significantly decreased albumin levels in malaria patients relative to non-malarial controls (*P* < 0.001, SMD = −2.23, 95% CI − 3.25 to − 1.20, *I*^2^: 98%, random effects model, 28 studies). SMD stands for standardized mean difference; CI stands for confidence interval; blue squares represent individual study effect estimates; the gray diamond represents the pooled effect estimate. Fourteen studies were observed to be outliers (asterisks)^26,27,32,34–36,38,39,43,47,52,58,60,61^.

The difference in albumin levels between malaria patients and non-malarial controls was pooled using the quantitative data from 28 studies^26–28,32–45,47–49,52,54,55,58–62^. The meta-analysis revealed markedly decreased albumin levels in malaria patients relative to the non-malarial controls (*P* < 0.001, SMD = −2.23, 95% CI − 3.25 to − 1.20), *I*^2^: 98%, random effects model, 28 studies, Fig. 3).

The meta-analysis results were heterogeneous (*I*^2^: 98%); therefore, meta-regression and subgroup analyses were carried out to investigate the possible origins of this variability. The meta-regression, which considered years of publication, design of the studies, continental distribution, demographic age groups, species of *Plasmodium*, clinical status (symptomatic vs. asymptomatic and severe vs. non-severe), diagnostic method for malaria, along with the types of blood samples used, revealed that none of these elements had a significant impact on the combined estimate (Table S4).

**Figure 4:**
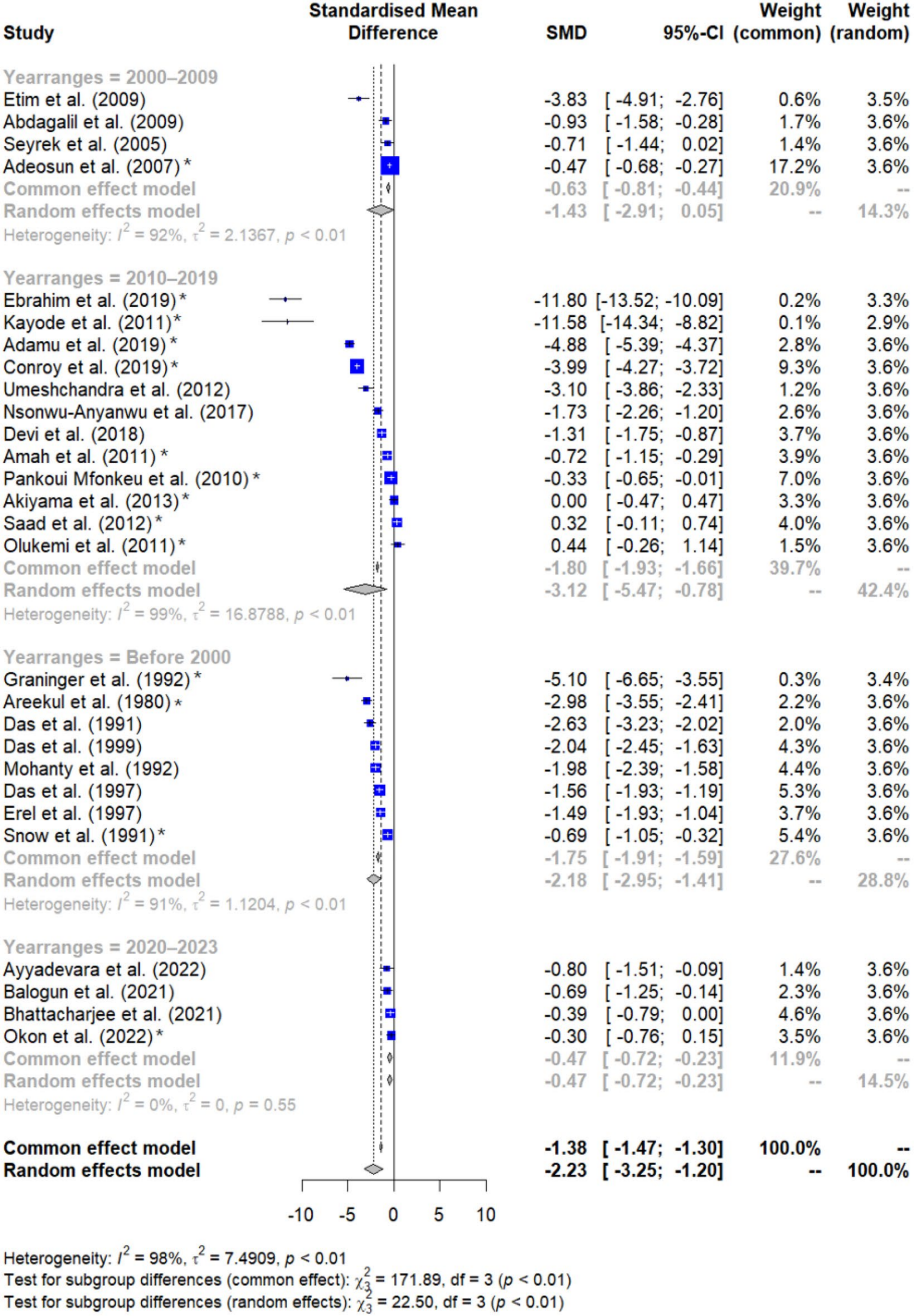
Forest plot displaying significantly decreased albumin levels in malaria patients relative to non-malarial controls stratified by publication years. SMD stands for standardized mean difference; CI stands for confidence interval; blue squares represent individual study effect estimates; the gray diamond represents the pooled effect estimate. Fourteen studies were observed to be outliers (asterisks)^26,27,32,34–36,38,39,43,47,52,58,60,61^.

**Figure 5:**
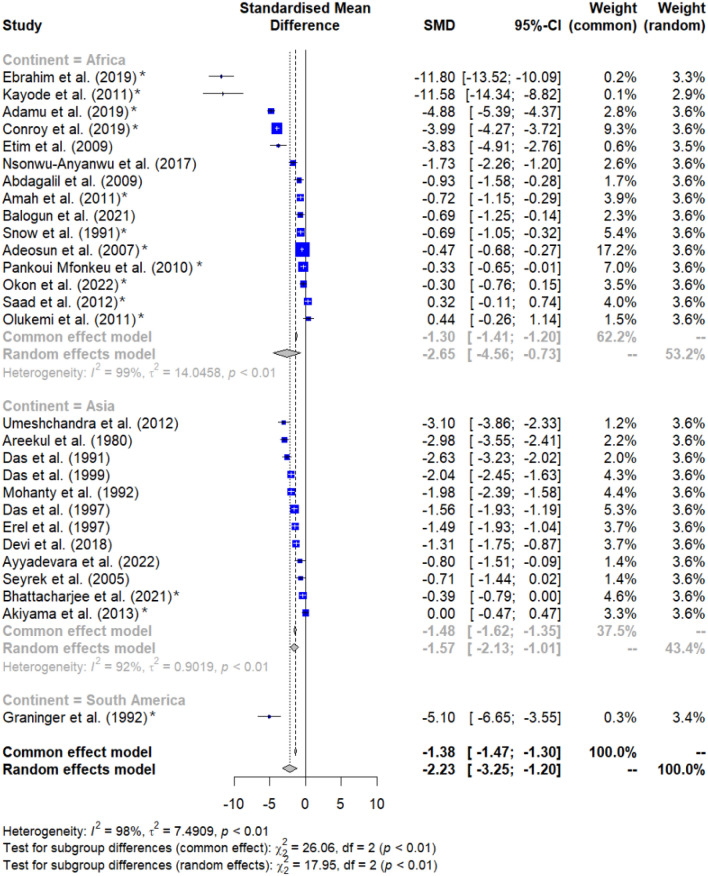
Forest plot displaying significantly decreased albumin levels in malaria patients relative to non-malarial controls stratified by continent. SMD stands for standardized mean difference; CI stands for confidence interval; blue squares represent individual study effect estimates; the gray diamond represents the pooled effect estimate. Fourteen studies were observed to be outliers (asterisks)^26,27,32,34–36,38,39,43,47,52,58,60,61^.

**Figure 6:**
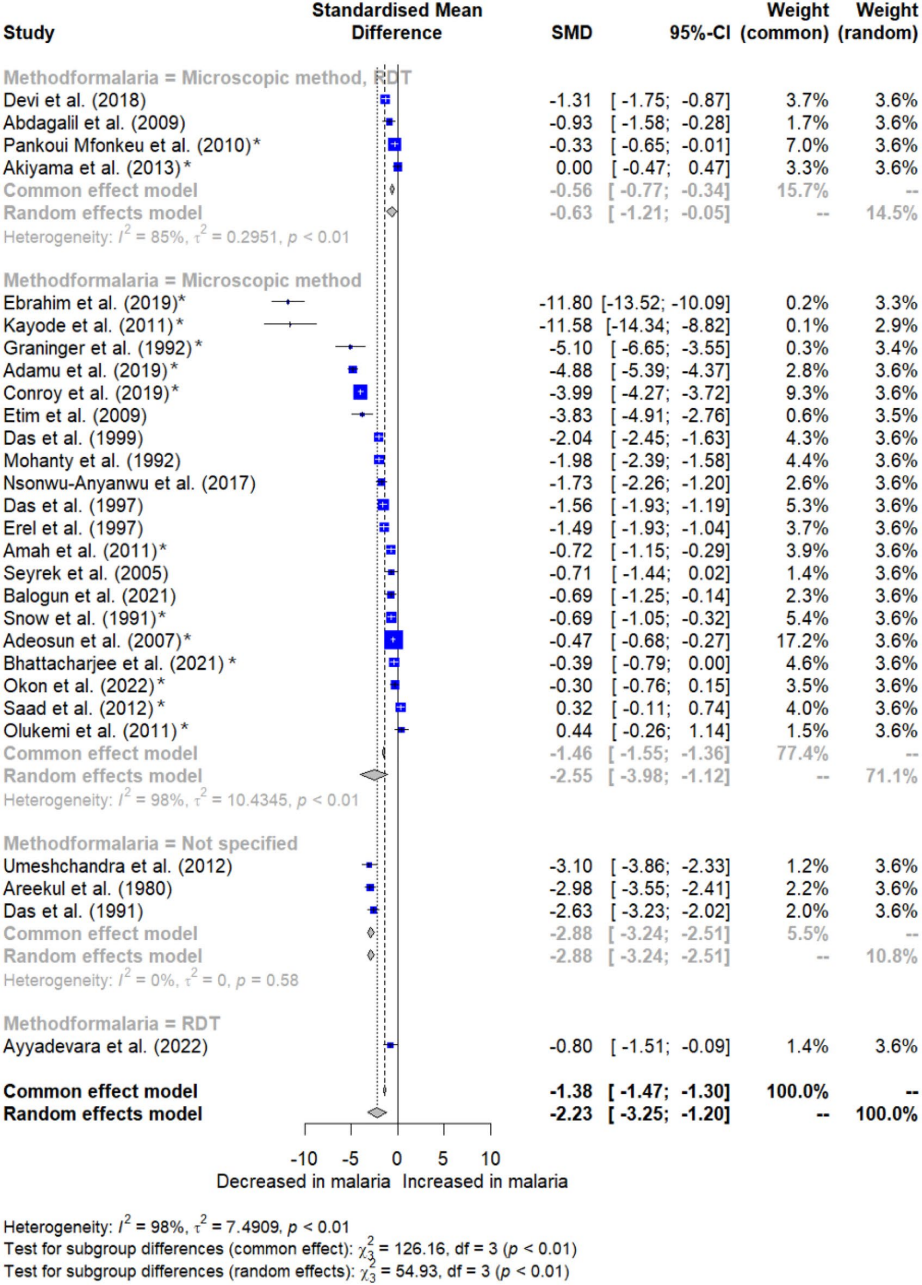
Forest plot displaying significantly decreased albumin levels in malaria patients relative to non-malarial controls stratified by methods for *Plasmodium* identification. SMD stands for standardized mean difference; CI stands for confidence interval; blue squares represent individual study effect estimates; the gray diamond represents the pooled effect estimate. Fourteen studies were observed to be outliers (asterisks)^26,27,32,34–36,38,39,43,47,52,58,60,61^.

Subsequent subgroup analyses revealed significant differences based on publication years (*P* < 0.001, Fig. 4), continent (*P* < 0.001, Fig. 5), and methods for *Plasmodium* identification (*P* < 0.001, Fig. 6). Specifically, studies conducted before 2000, between 2010 and 2019, and from 2020 to 2023 exhibited variations in albumin levels between malaria patients and non-malarial controls, whereas those from 2000 to 2009 did not show such a difference. Regarding continental differences, studies in Africa had a larger effect size (Hedges’ g = −2.646) compared to those in Asia (Hedges’ g = −1.574). For the diagnostic methods, studies using microscopy method alone revealed a greater effect size (Hedges’ g = −2.555) versus studies using a combination of microscopy and RDT methods (Hedges’ g = −0.629) (Table 3).

**Table 3:** Subgroup analysis of the difference in albumin levels between malaria patients and non-malarial controls.

Subgroup analyses	Test for subgroup differences (*P* value)	Hedges’ g [95% CI]	*I*^2^ (%)	Number of studies
Publication years	< 0.001			
2020–2023		− 0.474 [− 0.721 to − 0.227]	0.0	4
2010–2019		− 3.124 [− 5.467 to − 0.781]	98.7	12
2000–2009		− 1.431 [− 2.909 to 0.046]	91.9	4
Before 2000		− 2.179 [− 2.946 to − 1.412]	91.4	8
Study design	0.221			
Case–control study		− 2.220 [− 3.631 to − 0.809]	97.2	15
Cross-sectional study		− 1.780 [− 4.259 to 0.698]	94.8	8
Cohort study		− 2.880 [− 4.770 to − 0.990]	98.6	4
Quasi-experimental study		− 3.834 [− 4.913 to − 2.756]	N/A	1
Continent	< 0.001			
Africa		− 2.646 [− 4.558 to − 0.733]	98.6	15
Asia		− 1.574 [− 2.133 to − 1.014]	92.5	12
South America		− 5.100 [− 6.654 to − 3.546]	N/A	1
Age group	0.376			
Children		− 1.391 [− 2.266 to − 0.517]	98.6	8
Adults		− 1.818 [− 2.823 to − 0.813]	95.2	11
Children and adults		− 4.869 [− 9.212 to − 0.525]	98.7	6
Not specified		− 1.227 [− 2.135 to − 0.319]	93.4	3
*Plasmodium* species	0.133			
* P. falciparum*		− 2.471 [− 3.889 to − 1.054]	98.0	20
* P. vivax*		− 1.157 [− 1.910 to − 0.404]	68.5	2
* P. falciparum*/*P. vivax*		− 0.797 [− 1.509 to − 0.085]	N/A	1
Not specified		− 2.126 [− 3.728 to − 0.525]	98.1	5
Symptoms	0.001			
Symptomatic malaria		− 2.792 [− 4.410 to − 1.174]	98.1	17
Asymptomatic malaria		0.000 [− 0.469 to 0.469]		1
Symptomatic and asymptomatic malaria		− 1.123 [− 1.975 to − 0.272]	90.7	2
Not specified		− 1.710 [− 2.931 to − 0.488]	97.3	8
Methods for *Plasmodium* identification	< 0.001			
Microscopic method		− 2.555 [− 3.985 to − 1.125]	98.1	20
Microscopic method, RDT		− 0.629 [− 1.212 to − 0.046]	84.8	4
RDT		− 0.797 [− 1.509 to − 0.085]	N/A	1
Not specified		− 2.875 [− 3.239 to − 2.511]	0	3
Blood samples for albumin measurement	0.570			
Serum		− 2.192 [− 3.561 to − 0.822]	97.3	17
Plasma		− 1.634 [− 2.418 to − 0.849]	98.4	9
Not specified		− 6.105 [− 16.67 to 4.465]	98.2	2

**Figure 7:**
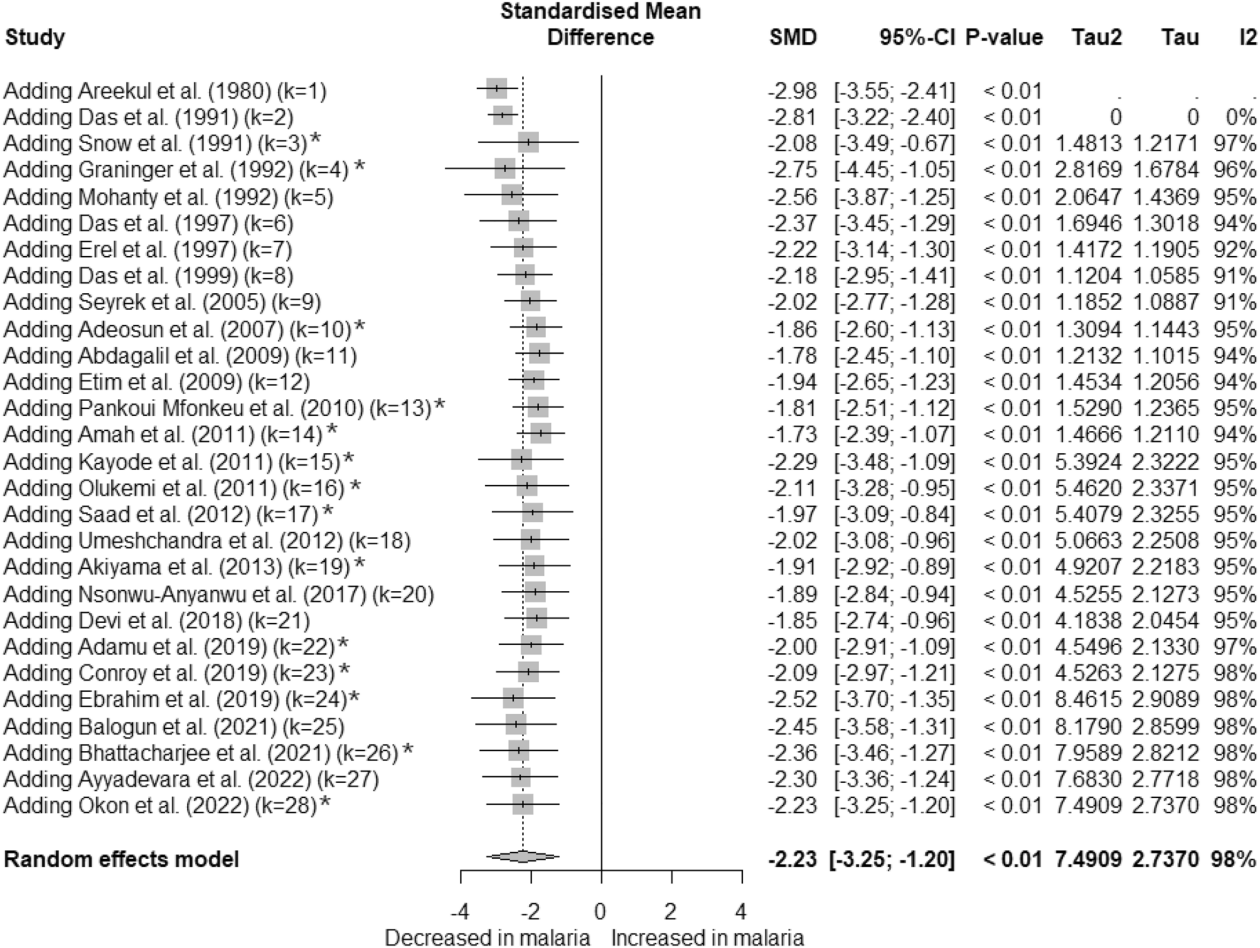
Cumulative meta-analysis forest plot depicting albumin level differences over time. This plot illustrates the significant changes in albumin levels as additional studies are included over time, each reinforcing the overall observed differences (*P* < 0.001). The standardized mean difference (SMD) was represented by gray squares, indicating the effect estimate of each study at the time of its publication. The pooled effect estimates across all studies are shown as a gray diamond. Fourteen studies were observed to be outliers (asterisks)^26,27,32,34–36,38,39,43,47,52,58,60,61^.

The cumulative meta-analysis was conducted to assess the evolving trend in albumin levels between malaria patients and non-malarial controls over time. The results demonstrated a significant difference that became more pronounced with the inclusion of each successive study (*P* < 0.001, Fig. 7).

**Figure 8:**
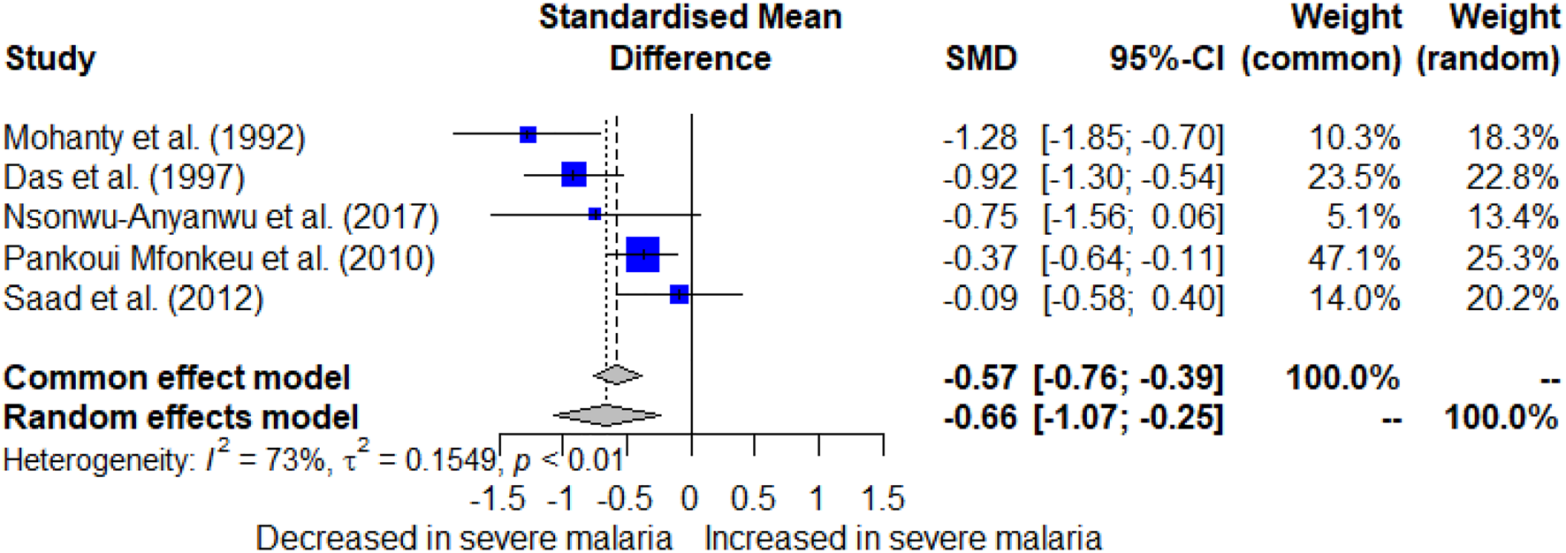
Forest plot of albumin level differences between patients experiencing severe malaria relative to those with less severe forms of the disease. This plot displays a significant decrease in albumin levels among patients experiencing severe malaria relative to those with less severe forms of the disease (*P* < 0.001, SMD: − 0.66, 95% CI − 1.07 to − 0.25, *I*^2^: 73%, 5 studies, random effects model). Blue squares represent the effect estimates of individual studies; the gray diamond indicates the pooled effect estimate.

The difference in albumin levels between patients experiencing severe malaria relative to those with less severe forms of the disease was pooled using the quantitative data from five studies^33,36,45,49,52^. The meta-analysis showed significantly decreased albumin levels in patients experiencing severe malaria relative to those with less severe forms of the disease (*P* < 0.001, SMD = −0.66, 95% CI − 1.07 to − 0.25), *I*^2^: 73%, random effects model, 5 studies, Fig. 8). Meta-regression and subgroup analyses could not be conducted due to the small number of studies involved.

**Figure 9:**
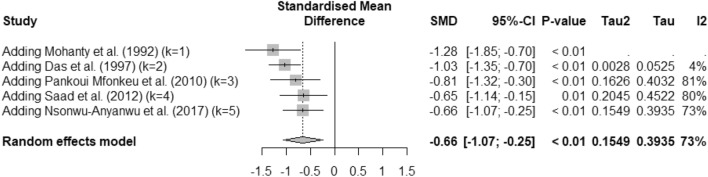
Forest plot illustrating changes in albumin levels over time. This plot indicates a significant difference in albumin levels between groups across various studies over time, with each subsequent study reinforcing the observed trend (*P* < 0.001). The gray squares represent the effect estimates of individual studies, with the size corresponding to the weight of the study in the meta-analysis. The horizontal lines through the squares indicate the 95% confidence intervals. The gray diamond represents the pooled effect estimate across all studies. SMD stands for standardized mean difference.

The cumulative meta-analysis aimed to assess the evolving trend in albumin levels between patients experiencing severe malaria relative to those with less severe forms of the disease over the years. The results showed a significant difference at each time point, reinforced by each additional study (*P* < 0.001, Fig. 9).

### Publication bias

**Figure 10:**
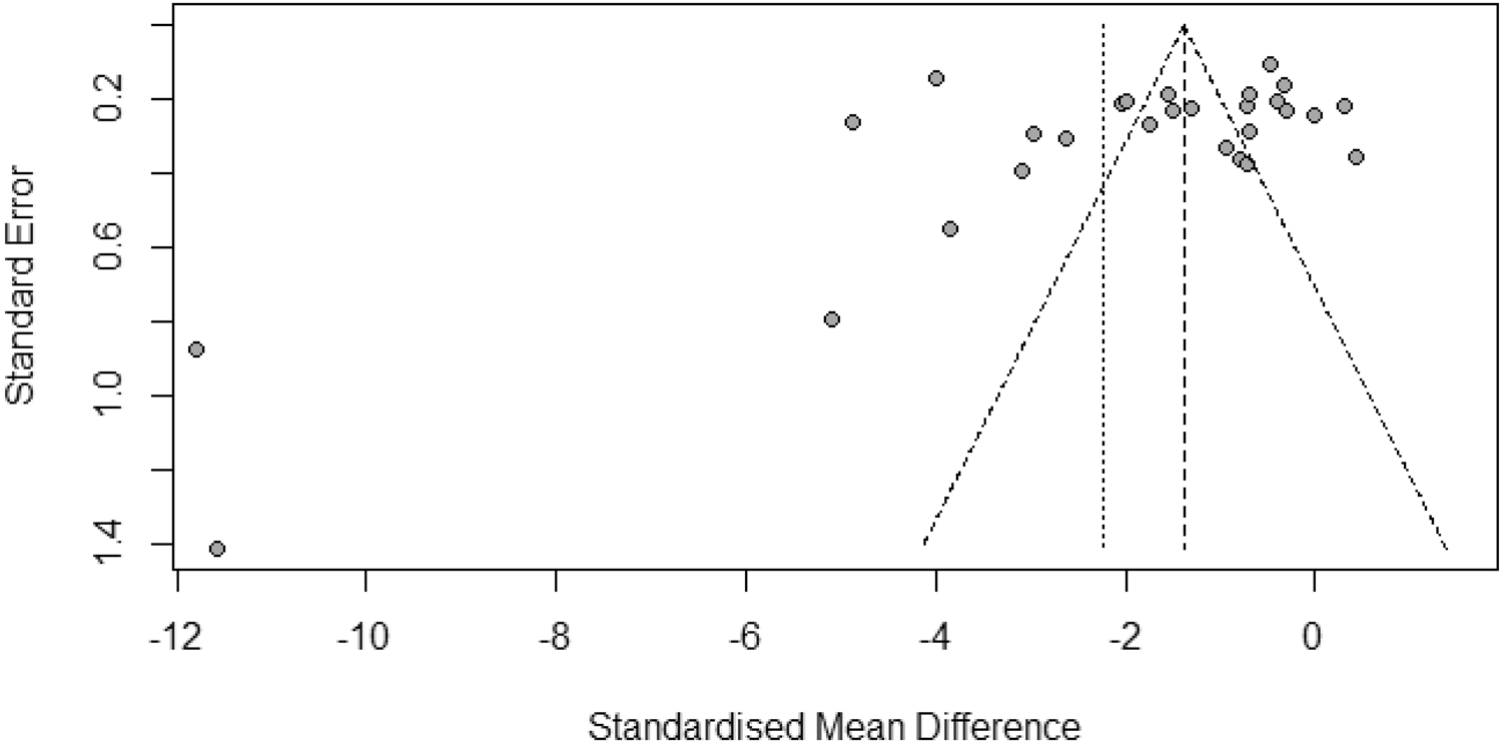
Funnel plot of effect estimates for albumin levels. This funnel plot shows an uneven distribution of effect size estimates (represented by grey dots) in relation to the middle line, which indicates potential publication bias or heterogeneity among the included studies.

The funnel plot of effect estimates indicated an asymmetrical distribution (Fig. 10), suggesting the potential for publication bias or other underlying heterogeneities among the included studies. Despite this initial observation, the linear regression test for funnel plot asymmetry did not reveal significant bias (*P* = 0.0811), implying that the absence of small studies might not be the sole contributor to the noted asymmetry. The trim-and-fill method has been applied, adjusting for potential publication bias by estimating and correcting for the number of missing studies. This adjustment indicated a notable reduction in albumin levels among malaria patients compared to non-malarial controls (*P* = 0.0376, SMD = −1.45, 95% CI − 2.81 to − 0.08), even when considering a high degree of heterogeneity (*I*^2^ = 97.8%). This result, derived from a random effects model incorporating 31 studies, underscores the robustness of findings despite the initial asymmetry.

### Influential analysis (sensitivity analysis)

An influence analysis was conducted to determine the effect of an individual study on the pooled results. For the variation in albumin levels among malaria patients in relation to non-malarial controls, the results showed that none of the included studies influenced the pooled results when an individual study was omitted and the meta-analysis was rerun (*P* < 0.01, Supplementary File 1). Similarly, for the difference in albumin levels in patients experiencing severe malaria relative to those with less severe forms of the disease, none of the studies included influenced the pooled results upon omission and rerunning of the meta-analysis (*P* < 0.05, Supplementary File 2).

### Outliers’ detection

For the variation in albumin levels among malaria patients in relation to non-malarial controls, fourteen studies were observed to be outliers^26,27,32,34–36,38,39,43,47,52,58,60,61^. After excluding these outliers from the meta-analysis, the results remained unchanged (*P* < 0.001, SMD = −1.812, 95% CI − 2.288 to − 1.335, *I*^2^: 86.5%, random effects model, 14 studies). In the analysis of albumin level differences between patients experiencing severe malaria relative to those with less severe forms of the disease, no outliers were detected using either fixed-effect or random-effects models.

### Power analysis

**Figure 11:**
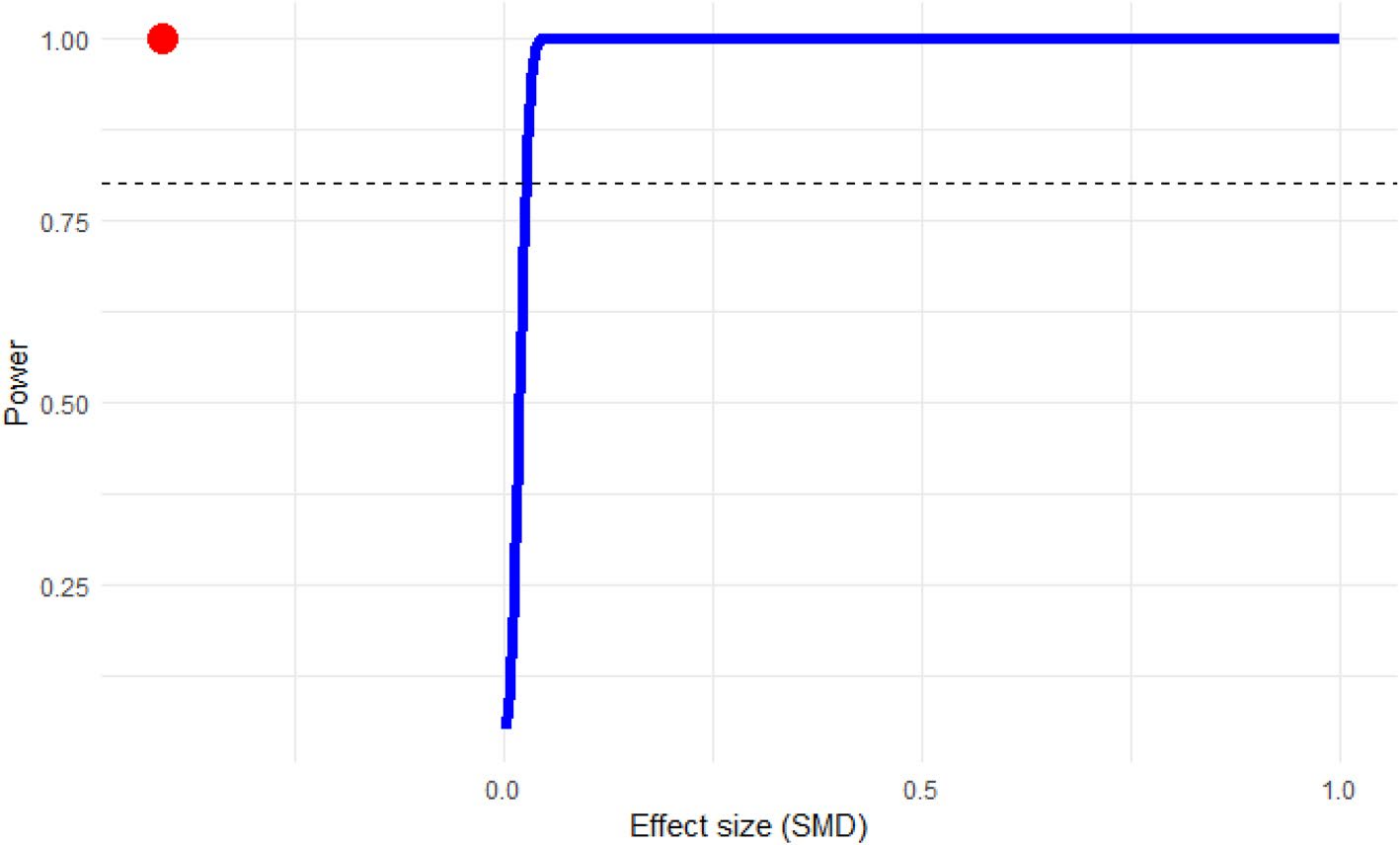
Power analysis for determining study adequacy in the meta-analysis of the difference in albumin levels between malaria patients and non-malarial controls. This analysis was conducted to ascertain the required number of studies for a robust meta-analysis. The power exceeded the threshold (dash line), indicating that the number of studies included was sufficient for a substantive meta-analysis. The red dot above the power line indicates that for the given effect size (SMD), the study has power exceeding the 0.80 threshold.

**Figure 12:**
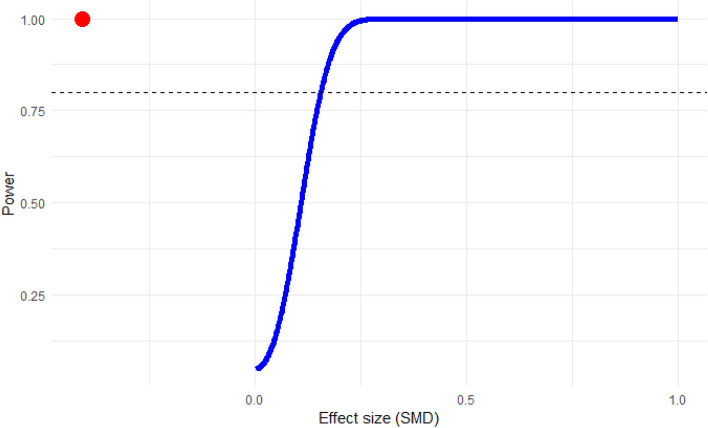
Power analysis for determining study adequacy in the meta-analysis of the difference in albumin levels between patients experiencing severe malaria relative to those with less severe forms of the disease. This analysis was conducted to ascertain the required number of studies for a robust meta-analysis. The power exceeded the threshold (dash line), indicating that the number of studies included was sufficient for a substantive meta-analysis. The red dot above the power line indicates that for the given effect size (SMD), the study has power exceeding the 0.80 threshold.

The power analysis was conducted to determine the number of studies required to perform a robust meta-analysis. The power threshold was set at 0.80 (80%). For the variation in albumin levels among malaria patients in relation to non-malarial controls, the results indicated that an adequate number of studies was included to draw a conclusion (Fig. 11). Similarly, the power analysis for the meta-analysis assessing the difference in albumin levels between patients experiencing severe malaria relative to those with less severe forms of the disease also demonstrated that a sufficient number of studies were included (Fig. 12).

## Discussion

The thematic synthesis of 37 studies, along with subsequent meta-analyses, presents compelling evidence regarding albumin levels in malaria patients. Notably, there was variation in the albumin levels observed in malaria patients in relation to non-malarial controls, influenced by geographical locations and malaria severity. The findings showed that albumin levels in malaria patients varied based on geographical location. In African studies, several reports^34,37,38,49,52,58,61^ did not observe any difference in albumin levels between malaria patients in relation to non-malarial controls, while a similar number of studies^26,29,32,39,42,47,62^ reported a decrease. In contrast, most Asian studies^28,33,40,41,44,45,48,54,55,57,59^ reported decreased albumin levels in malaria patients. Although the thematic synthesis indicated no notable disparity in albumin levels between malaria patients and non-malarial controls in African studies, the meta-analysis suggested a more substantial effect size in albumin level differences in this region. This discrepancy might be attributed to the meta-analysis, which, by quantitatively synthesizing data, could detect subtle differences not evident in the qualitative thematic synthesis. Overall, the meta-analysis results indicated a significant decrease in albumin levels in malaria patients compared to non-malarial controls, suggesting an overall trend. However, discrepancies among individual studies might be explained by differences in some characteristics of included studies, as suggested by the subgroup analysis. Although the meta-regression analysis did not identify any of the variables as significant contributors to the observed heterogeneity in the combined estimate, the subgroup analyses, which grouped studies based on specific characteristics for a more focused comparison, revealed significant differences in effect sizes associated with publication years, continent, and methods for *Plasmodium* identification. This discrepancy between the meta-regression and subgroup analysis outcomes can be attributed to varying analytical approaches. While meta-regression assessed the influence of covariates on the effect size across the entire dataset, subgroup analysis examines the effect sizes within defined categories of these covariates.

Concerning the publication years, the cumulative meta-analysis revealed that trends did not alter the results of the meta-analysis significantly. Subgroup analysis, however, showed decreased albumin levels among malaria patients in the studies conducted before 2000 (Hedges’ g = −2.179), between 2010 and 2019 (Hedges’ g = −3.124), and from 2020 to 2023 (Hedges’ g = −0.474). Studies from 2000 to 2009 demonstrated comparable albumin levels between malaria patients and non-malarial controls (Hedges’ g = −1.431). These differences may reflect the impact of various factors on meta-analysis results, such as genetic factors, nutritional status, changes in clinical management, the effectiveness of malaria control programs, and drug resistance patterns. In terms of continental differences, the subgroup analysis revealed a decrease in albumin levels in malaria patients across all continents (Africa, Asia, South America), with more pronounced decreases in African studies (Hedges’ g = −2.646) than in Asian studies (Hedges’ g = −1.574). This may highlight regional differences in genetic factors, nutritional status, healthcare access, or immune responses to *Plasmodium* infections. Regarding *Plasmodium* detection methods, subgroup analysis showed a decrease in albumin levels across all methods. More pronounced decreases were noted in studies using microscopy alone (Hedges’ g = −2.555) compared to those using a combination of microscopy and RDTs (Hedges’ g = −0.629). Despite the overall finding of decreased albumin levels in malaria patients, some studies^34,37,38,49,52,58,61^ found no change, possibly because albumin levels had not yet had time to decrease in acute cases^43^.

Focusing on disease severity, systematic reviews, and meta-analyses confirmed that severe malaria cases consistently had lower albumin levels compared to non-severe cases across continents, suggesting albumin as a potential biomarker for disease severity. This could aid in clinical assessment and treatment decisions. The findings align with previous studies that associated albumin levels below 35 g/L with severe malaria require intensive care^53^. Hepatic dysfunction, associated with severe malaria, may be responsible for progressively lower albumin levels, with *Plasmodium* infections increasing the transcapillary escape rate of albumin, including renal losses^34,46^. Another recent study suggested that increased capillary permeability was associated with serum albumin levels, correlating with disease severity and respiratory complications in patients with imported falciparum malaria^31^. Albumin, a plentiful circulating antioxidant^63,64^, plays a unique role due to its multiple ligand-binding capacity and free radical-capturing properties^65^. It also helps protect cells against oxidative stress by regulating cellular glutathione levels (GSH), with albumin catabolism providing sulfur-containing amino acids for GSH synthesis in cells^66^. Therefore, decreased albumin levels in malaria patients could lead to severe oxidative stress and worsen clinical outcomes. Contrarily, a previous study found increased albumin levels in patients with severe malaria, suggesting a compensatory effect against oxidative stress^30^. Following hospital treatment, serum albumin levels increased in both severe and uncomplicated malaria patients^67^, potentially attributable to improved clinical conditions or reduced capillary permeability post-treatment.

Despite the robust findings from the sensitivity analysis, which affirmed the stability of the combined outcomes by showing that no individual study excessively swayed the results. The power analysis confirmed that the number of studies included in the meta-analyses is adequate, supporting the conclusions' validity, but certain limitations exist. The variation in albumin levels across studies emphasizes the importance of considering local context when interpreting these results. Additionally, the high heterogeneity in some analyses suggests that other unmeasured factors may influence albumin levels in malaria patients. The assessment of publication bias via funnel plot and the trim-and-fill method indicated that the lack of small studies did not significantly influence the asymmetry observed in the funnel plot. The influence of co-infections, nutritional status, and socioeconomic factors should be considered in future studies.

The systematic review and meta-analysis offer significant implications for both clinical practice and research. Clinicians can leverage albumin levels to evaluate the severity of malaria and contemplate additional support for patients with lower levels. The timing of albumin measurement concerning the clinical course of malaria is pivotal for its utilization as a prognostic tool or an indicator of developing severity. Albumin levels might serve as predictive indicators for severe malaria if measured at multiple time points to discern whether early changes precede the onset of severe symptoms. For example, a previous study demonstrated a drop in albumin levels among patients with uncomplicated malaria who progressed to severe malaria during treatment^68^. Therefore, measuring albumin levels upon initial presentation and monitoring them throughout treatment could prove invaluable, as significant drops may signal the necessity for escalated care or closer monitoring. Moreover, awareness of a patient's risk for severe malaria would significantly influence clinical management. If albumin is validated as a predictor of severity, its measurement could become a routine aspect of assessing malaria patients. Clinicians could stratify patients based on their risk of developing severe malaria, enabling early intervention, and potentially improving outcomes. Notably, albumin has been employed as an adjunctive therapy in children with severe malaria, resulting in reduced mortality rates^69,70^. Albumin infusion is suggested to enhance microcirculation, correct hypoglycemia, and reduce lactic acidosis in patients with severe malaria^71^.

## Conclusion

Overall, this study affirmed that malaria infection was associated with decreased albumin levels, with more significant impacts noted in severe instances of the disease. These findings emphasized the role of albumin as a potential marker for malaria severity and underscored the need for personalized patient care. Given the affordability and accessibility of albumin testing, even in resource-limited settings, it could be a viable biomarker for assessing the severity of endemic as well as imported malaria.

### Supplementary Information

Supplementary Information 1.

Supplementary Information 2.

Supplementary Table S1.

Supplementary Table S2.

Supplementary Table S3.

Supplementary Table S4.

## Data Availability

All data relating to the present study are available in this manuscript, Table S1, Table S2, Table S3, Table S4 files.
