# Supplementary material for: Albumin levels in malaria patients: a systematic review and meta-analysis of their association with disease severity
Source: Sci Rep. 2024 May 3;14:10185. doi: 10.1038/s41598-024-60644-z (PMC11068903; doi:10.1038/s41598-024-60644-z)
Supplement: Supplementary file 1 — Supplementary Information 1. [file 41598_2024_60644_MOESM1_ESM.pdf]

Albumin levels in malaria patients: A systematic review and meta-analysis of their association with disease severity

Saruda Kuraeiad[1], Kwuntida Uthaisar Kotepui[1], Aongart Mahittikorn[2*], Frederick Ramirez Masangkay[3], Polrat Wilairatana[4], Apiporn Thinkhamrop Suwannatrai[5], Kavin Thinkhamrop[6], Kinley Wangdi[7], Manas Kotepui[1*]

[1]Medical Technology, School of Allied Health Sciences, Walailak University, Tha Sala, Nakhon Si Thammarat 80160, Thailand

[2]Department of Protozoology, Faculty of Tropical Medicine, Mahidol University, Bangkok 10400, Thailand

[3]Department of Medical Technology, Faculty of Pharmacy, University of Santo Tomas, Manila 1008, Philippines

[4]Department of Clinical Tropical Medicine, Faculty of Tropical Medicine, Mahidol University, Bangkok 10400, Thailand

[5]Department of Parasitology, Faculty of Medicine, Khon Kaen University, Khon Kaen 40002, Thailand

[6]Faculty of Public Health, Khon Kaen University, Khon Kaen 40002, Thailand

[7]QIMR Medical Research Institute, 300 Herston Road, Herston QLD 4006 Australia

*Corresponding authors

Saruda Kuraeiad: saruda.ku@wu.ac.th

Kwuntida Uthaisar Kotepui: kwuntida.ut@wu.ac.th

Aongart Mahittikorn: aongart.mah@mahidol.ac.th

Frederick Ramirez Masangkay: frmasangkay@ust.edu.ph

Polrat Wilairatana: polrat.wil@mahidol.ac.th

Apiporn Thinkhamrop Suwannatrai: apiporn@kku.ac.th

Kavin Thinkhamrop: kavith@kku.ac.th

Kinley Wangdi: kinley.wangdi@qimrberghofer.edu.au

Manas Kotepui manas.ko@wu.ac.th, Tel+ :.66954392469

Supplementary File 1. Influential analysis (sensitivity analysis) for the difference in albumin levels between malaria patients and non-malarial controls.

```
Influential analysis (random effects model)

                                                SMD              95%-CI  p-val
ue   tau^2     tau
Omitting Abdagalil et al. (2009)            -2.2781 [-3.3417; -1.2145] < 0.00
01  7.7750  2.7884
Omitting Adamu et al. (2019)                -2.1266 [-3.1703; -1.0828] < 0.00
01  7.4796  2.7349
Omitting Adeosun et al. (2007)              -2.2954 [-3.3550; -1.2358] < 0.00
01  7.7123  2.7771
Omitting Akiyama et al. (2013)              -2.3117 [-3.3653; -1.2581] < 0.00
01  7.6241  2.7612
Omitting Amah et al. (2011)                 -2.2860 [-3.3481; -1.2239] < 0.00
01  7.7500  2.7839
Omitting Areekul et al. (1980)              -2.2021 [-3.2683; -1.1359] < 0.00
01  7.8131  2.7952
Omitting Ayyadevara et al. (2022)           -2.2828 [-3.3452; -1.2204] < 0.00
01  7.7582  2.7854
Omitting Balogun et al. (2021)              -2.2868 [-3.3485; -1.2251] < 0.00
01  7.7454  2.7831
Omitting Bhattacharjee et al. (2021)        -2.2980 [-3.3566; -1.2393] < 0.00
01  7.6987  2.7747
Omitting Conroy et al. (2019)               -2.1618 [-3.2193; -1.1044] < 0.00
01  7.6801  2.7713
Omitting Das et al. (1991)                  -2.2155 [-3.2831; -1.1478] < 0.00
01  7.8349  2.7991
Omitting Das et al. (1999)                  -2.2374 [-3.3061; -1.1688] < 0.00
01  7.8480  2.8014
Omitting Das et al. (1997)                  -2.2556 [-3.3232; -1.1880] < 0.00
01  7.8321  2.7986
Omitting Devi et al. (2018)                 -2.2647 [-3.3311; -1.1984] < 0.00
01  7.8142  2.7954
Omitting Ebrahim et al. (2019)              -1.8448 [-2.6061; -1.0836] < 0.00
01  3.9297  1.9823
Omitting Erel et al. (1997)                 -2.2581 [-3.3253; -1.1908] < 0.00
01  7.8273  2.7977
Omitting Etim et al. (2009)                 -2.1704 [-3.2288; -1.1119] < 0.00
01  7.7054  2.7759
Omitting Graninger et al. (1992)            -2.1256 [-3.1660; -1.0852] < 0.00
01  7.4511  2.7297
Omitting Kayode et al. (2011)               -1.9322 [-2.8055; -1.0589] < 0.00
01  5.2422  2.2896
Omitting Mohanty et al. (1992)              -2.2397 [-3.3083; -1.1711] < 0.00
01  7.8473  2.8013
Omitting Nsonwu-Anyanwu et al. (2017)       -2.2491 [-3.3170; -1.1811] < 0.00
01  7.8389  2.7998
Omitting Okon et al. (2022)                 -2.3010 [-3.3586; -1.2435] < 0.00
01  7.6830  2.7718
Omitting Olukemi et al. (2011)              -2.3262 [-3.3729; -1.2796] < 0.00
01  7.5248  2.7431
```

```
Omitting Pankoui Mfonkeu et al. (2010)   -2.3003 [-3.3583; -1.2423] < 0.00
01  7.6880  2.7727
Omitting Saad et al. (2012)              -2.3229 [-3.3716; -1.2742] < 0.00
01  7.5524  2.7482
Omitting Seyrek et al. (2005)            -2.2857 [-3.3474; -1.2241] < 0.00
01  7.7469  2.7833
Omitting Snow et al. (1991)              -2.2874 [-3.3492; -1.2256] < 0.00
01  7.7454  2.7830
Omitting Umeshchandra et al. (2012)      -2.1977 [-3.2629; -1.1325] < 0.00
01  7.8002  2.7929

Pooled estimate                          -2.2263 [-3.2517; -1.2009] < 0.00
01  7.4909  2.7370
                                            I^2
Omitting Abdagalil et al. (2009)          97.8%
Omitting Adamu et al. (2019)              97.3%
Omitting Adeosun et al. (2007)            97.6%
Omitting Akiyama et al. (2013)            97.7%
Omitting Amah et al. (2011)               97.7%
Omitting Areekul et al. (1980)            97.7%
Omitting Ayyadevara et al. (2022)         97.8%
Omitting Balogun et al. (2021)            97.8%
Omitting Bhattacharjee et al. (2021)      97.7%
Omitting Conroy et al. (2019)             96.7%
Omitting Das et al. (1991)                97.7%
Omitting Das et al. (1999)                97.7%
Omitting Das et al. (1997)                97.8%
Omitting Devi et al. (2018)               97.8%
Omitting Ebrahim et al. (2019)            97.5%
Omitting Erel et al. (1997)               97.8%
Omitting Etim et al. (2009)               97.7%
Omitting Graninger et al. (1992)          97.7%
Omitting Kayode et al. (2011)             97.7%
Omitting Mohanty et al. (1992)            97.7%
Omitting Nsonwu-Anyanwu et al. (2017)     97.8%
Omitting Okon et al. (2022)               97.7%
Omitting Olukemi et al. (2011)            97.7%
Omitting Pankoui Mfonkeu et al. (2010)    97.7%
Omitting Saad et al. (2012)               97.6%
Omitting Seyrek et al. (2005)             97.8%
Omitting Snow et al. (1991)               97.7%
Omitting Umeshchandra et al. (2012)       97.7%

Pooled estimate                           97.7%

Details on meta-analytical method:
- Inverse variance method
- Restricted maximum-likelihood estimator for tau^2
```
