# Supplementary material for: Albumin levels in malaria patients: a systematic review and meta-analysis of their association with disease severity
Source: Sci Rep. 2024 May 3;14:10185. doi: 10.1038/s41598-024-60644-z (PMC11068903; doi:10.1038/s41598-024-60644-z)
Supplement: Supplementary file 3 — Supplementary Table S1. [file 41598_2024_60644_MOESM3_ESM.docx]

**Albumin levels in malaria patients: A systematic review and meta-analysis of their association with disease severity**

Saruda Kuraeiad^1^, Kwuntida Uthaisar Kotepui^1^, Aongart Mahittikorn^2*^, Frederick Ramirez Masangkay^3^, Polrat Wilairatana^4^, Apiporn Thinkhamrop Suwannatrai^5^, Kavin Thinkhamrop^6^, Kinley Wangdi^7^, Manas Kotepui^1*^

^1^Medical Technology, School of Allied Health Sciences, Walailak University, Tha Sala, Nakhon Si Thammarat 80160, Thailand

^2^Department of Protozoology, Faculty of Tropical Medicine, Mahidol University, Bangkok 10400, Thailand

^3^Department of Medical Technology, Faculty of Pharmacy, University of Santo Tomas, Manila 1008, Philippines

^4^Department of Clinical Tropical Medicine, Faculty of Tropical Medicine, Mahidol University, Bangkok 10400, Thailand

^5^Department of Parasitology, Faculty of Medicine, Khon Kaen University, Khon Kaen 40002, Thailand

^6^Faculty of Public Health, Khon Kaen University, Khon Kaen 40002, Thailand

^7^QIMR Medical Research Institute, 300 Herston Road, Herston QLD 4006 Australia

*Corresponding authors

Saruda Kuraeiad: saruda.ku@wu.ac.th

Kwuntida Uthaisar Kotepui: [kwuntida.ut@wu.ac.th](mailto:kwuntida.ut@wu.ac.th)

Aongart Mahittikorn: [aongart.mah@mahidol.ac.th](mailto:aongart.mah@mahidol.ac.th)

Frederick Ramirez Masangkay: frmasangkay@ust.edu.ph

Polrat Wilairatana: [polrat.wil@mahidol.ac.th](mailto:polrat.wil@mahidol.ac.th)

Apiporn Thinkhamrop Suwannatrai: [apiporn@kku.ac.th](mailto:apiporn@kku.ac.th)

Kavin Thinkhamrop: kavith@kku.ac.th

Kinley Wangdi: [kinley.wangdi@qimrberghofer.edu.au](mailto:kinley.wangdi@qimrberghofer.edu.au)

Manas Kotepui [manas.ko@wu.ac.th](mailto:manas.ko@wu.ac.th), Tel.: +66954392469

**Table S1. Search terms**

**General keywords**

albumin AND (malaria OR plasmodium OR “Plasmodium Infection“ OR “Remittent Fever“ OR “Marsh Fever“ OR Paludism)

PubMed 11 October 2023

| No. | Key concept | Search terms | Results |
| --- | --- | --- | --- |
| 1. | Albumin | albumin[Text Word] OR albumin[MeSH Terms] | 300,963 |
| 2. | Malaria | malaria[Text Word] OR malaria[MeSH Terms] OR plasmodium[Text Word] OR “Plasmodium Infection“[Text Word] OR “Remittent Fever“[Text Word] OR “Marsh Fever“[Text Word] OR Paludism[Text Word] | 119,715 |
| 3. | #1 AND #2 | (albumin[Text Word] OR albumin[MeSH Terms]] OR hematoidin[Text Word] OR hematoidin[MeSH Terms]) AND (malaria[Text Word] OR malaria[MeSH Terms] OR plasmodium[Text Word] OR “Plasmodium Infection“[Text Word] OR “Remittent Fever“[Text Word] OR “Marsh Fever“[Text Word] OR Paludism[Text Word]) | 616 |

Embase 11 October 2023

| No. | Key concept | Search terms | Results |
| --- | --- | --- | --- |
| 1. | Albumin | albumin:ti,ab,kw,de OR albumin/exp | 346,538 |
| 2. | Malaria | malaria:ti,ab,kw,de OR plasmodium:ti,ab,kw,de OR ‘Remittent Fever’:ti,ab,kw,de OR ‘Marsh Fever’:ti,ab,kw,de OR Paludism:ti,ab,kw,de OR malaria/exp | 158,234 |
| 3. | 1 AND 2 | (albumin:ti,ab,kw,de OR albumin/exp) AND (malaria:ti,ab,kw,de OR plasmodium:ti,ab,kw,de OR ‘Remittent Fever’:ti,ab,kw,de OR ‘Marsh Fever’:ti,ab,kw,de OR Paludism:ti,ab,kw,de OR malaria/exp) | 843 |

Scopus 11 October 2023

| No. | Key concept | Search terms | Results |
| --- | --- | --- | --- |
| 1. | Albumin | TITLE-ABS-KEY (albumin) | 348,540 |
| 2. | Malaria | TITLE-ABS-KEY ( malaria OR plasmodium OR "plasmodium infection" OR "remittent fever" OR "marsh fever" OR paludism ) | 159,103 |
| 3. | 1 AND 2 | ( TITLE-ABS-KEY (albumin) AND ( TITLE-ABS-KEY ( malaria OR plasmodium OR "plasmodium infection" OR "remittent fever" OR "marsh fever" OR paludism ) | 840 |

Ovid 11 October 2023

| No. | Key concept | Search terms | Results |
| --- | --- | --- | --- |
| 1. | Albumin AND Malaria | albumin AND (malaria OR plasmodium OR "Plasmodium Infection" OR "Remittent Fever" OR "Marsh Fever" OR Paludism){Including Limited Related Terms}  Filter: ovid full text available and articles with abstracts and original articles | 263 |

MEDLINE 11 October 2023

| No. | Key concept | Search terms | Results |
| --- | --- | --- | --- |
| 1. | Albumin AND Malaria | albumin AND (malaria OR plasmodium OR "Plasmodium Infection" OR "Remittent Fever" OR "Marsh Fever" OR Paludism) | 421 |

Google Scholar 11 October 2023

| No. | Key concept | Search terms | Results |
| --- | --- | --- | --- |
| 1. | Albumin AND Malaria | Albumin AND Malaria | Screening only the first 200 articles |
