# Supplementary material for: Albumin levels in malaria patients: a systematic review and meta-analysis of their association with disease severity
Source: Sci Rep. 2024 May 3;14:10185. doi: 10.1038/s41598-024-60644-z (PMC11068903; doi:10.1038/s41598-024-60644-z)
Supplement: Supplementary file 6 — Supplementary Table S4. [file 41598_2024_60644_MOESM6_ESM.docx]

**Albumin levels in malaria patients: A systematic review and meta-analysis of their association with disease severity**

Saruda Kuraeiad^1^, Kwuntida Uthaisar Kotepui^1^, Aongart Mahittikorn^2*^, Frederick Ramirez Masangkay^3^, Polrat Wilairatana^4^, Apiporn Thinkhamrop Suwannatrai^5^, Kavin Thinkhamrop^6^, Kinley Wangdi^7^, Manas Kotepui^1*^

^1^Medical Technology, School of Allied Health Sciences, Walailak University, Tha Sala, Nakhon Si Thammarat 80160, Thailand

^2^Department of Protozoology, Faculty of Tropical Medicine, Mahidol University, Bangkok 10400, Thailand

^3^Department of Medical Technology, Faculty of Pharmacy, University of Santo Tomas, Manila 1008, Philippines

^4^Department of Clinical Tropical Medicine, Faculty of Tropical Medicine, Mahidol University, Bangkok 10400, Thailand

^5^Department of Parasitology, Faculty of Medicine, Khon Kaen University, Khon Kaen 40002, Thailand

^6^Faculty of Public Health, Khon Kaen University, Khon Kaen 40002, Thailand

^7^QIMR Medical Research Institute, 300 Herston Road, Herston QLD 4006 Australia

*Corresponding authors

Saruda Kuraeiad: saruda.ku@wu.ac.th

Kwuntida Uthaisar Kotepui: [kwuntida.ut@wu.ac.th](mailto:kwuntida.ut@wu.ac.th)

Aongart Mahittikorn: [aongart.mah@mahidol.ac.th](mailto:aongart.mah@mahidol.ac.th)

Frederick Ramirez Masangkay: frmasangkay@ust.edu.ph

Polrat Wilairatana: [polrat.wil@mahidol.ac.th](mailto:polrat.wil@mahidol.ac.th)

Apiporn Thinkhamrop Suwannatrai: [apiporn@kku.ac.th](mailto:apiporn@kku.ac.th)

Kavin Thinkhamrop: kavith@kku.ac.th

Kinley Wangdi: [kinley.wangdi@qimrberghofer.edu.au](mailto:kinley.wangdi@qimrberghofer.edu.au)

Manas Kotepui [manas.ko@wu.ac.th](mailto:manas.ko@wu.ac.th), Tel.: +66954392469

**Table S4. Meta-regression analysis of covariates on the difference in albumin levels between patients with malaria and uninfected controls**

| **Covariates** | ***P* value** | **tau^2^** | ***I^2^* (%)** | **R-squared (%)** | **Number of studies** |
| --- | --- | --- | --- | --- | --- |
| Publication years | 0.9387 | 7.8453 | 99.30 | 0.00 | 28 |
| Study design | 0.8535 | 8.2362 | 99.31 | 0.00 | 28 |
| Continent | 0.3895 | 7.5978 | 99.30 | 0.00 | 28 |
| Age group | 0.1078 | 6.9242 | 99.20 | 7.57 | 28 |
| *Plasmodium* species | 0.8832 | 8.4139 | 99.37 | 0.00 | 28 |
| Clinical status (symptomatic vs. asymptomatic) | 0.6279 | 8.0547 | 99.31 | 0.00 | 28 |
| Clinical status (severe vs. non-severe) | 0.8903 | 9.0370 | 99.31 | 0.00 | 28 |
| Diagnostic method for malaria | 0.5942 | 7.9446 | 99.33 | 0.00 | 28 |
| Types of blood samples | 0.2073 | 7.4382 | 99.27 | 0.70 | 28 |
